# Supplementary material for: Pyruvate Dehydrogenase Kinase 1 inhibition mediated oxidative phosphorylation enhancement in cartilage promotes osteoarthritis progression
Source: BMC Musculoskelet Disord. 2023 Jul 20;24:597. doi: 10.1186/s12891-023-06585-6 (PMC10357736; doi:10.1186/s12891-023-06585-6)
Supplement: Supplementary file 1 — Additional file 1. [file 12891_2023_6585_MOESM1_ESM.zip › BP.Gsea.1653623667859/GOBP_DICARBOXYLIC_ACID_METABOLIC_PROCESS.html]

Details for gene set GOBP\_DICARBOXYLIC\_ACID\_METABOLIC\_PROCESS[GSEA]

|  || Dataset | 55457\_collapsed\_to\_symbols.cls.cls #OA\_versus\_normal.cls.cls #OA\_versus\_normal\_repos |
| Phenotype | cls.cls#OA\_versus\_normal\_repos |
| Upregulated in class | OA |
| GeneSet | GOBP\_DICARBOXYLIC\_ACID\_METABOLIC\_PROCESS |
| Enrichment Score (ES) | 0.3609856 |
| Normalized Enrichment Score (NES) | 1.5759307 |
| Nominal p-value | 0.010335918 |
| FDR q-value | 0.28878298 |
| FWER p-Value | 1.0 |
Table: GSEA Results Summary

  

Fig 1: Enrichment plot: GOBP\_DICARBOXYLIC\_ACID\_METABOLIC\_PROCESS      
 Profile of the Running ES Score & Positions of GeneSet Members on the Rank Ordered List

  

| SYMBOL | TITLE | RANK IN GENE LIST | RANK METRIC SCORE | RUNNING ES | CORE ENRICHMENT || 1 | FOLR1 | folate receptor alpha [Source:HGNC Symbol;Acc:HGNC:3791] | 8 | 1.202 | 0.0439 | Yes |
| 2 | GOT1 | glutamic-oxaloacetic transaminase 1 [Source:HGNC Symbol;Acc:HGNC:4432] | 59 | 0.974 | 0.0761 | Yes |
| 3 | MDH1 | malate dehydrogenase 1 [Source:HGNC Symbol;Acc:HGNC:6970] | 271 | 0.772 | 0.0883 | Yes |
| 4 | GRHPR | glyoxylate and hydroxypyruvate reductase [Source:HGNC Symbol;Acc:HGNC:4570] | 363 | 0.720 | 0.1079 | Yes |
| 5 | ATIC | 5-aminoimidazole-4-carboxamide ribonucleotide formyltransferase/IMP cyclohydrolase [Source:HGNC Symbol;Acc:HGNC:794] | 401 | 0.706 | 0.1312 | Yes |
| 6 | MDH2 | malate dehydrogenase 2 [Source:HGNC Symbol;Acc:HGNC:6971] | 615 | 0.618 | 0.1375 | Yes |
| 7 | KMO | kynurenine 3-monooxygenase [Source:HGNC Symbol;Acc:HGNC:6381] | 694 | 0.595 | 0.1535 | Yes |
| 8 | PCK2 | "phosphoenolpyruvate carboxykinase 2, mitochondrial [Source:HGNC Symbol;Acc:HGNC:8725]" | 759 | 0.579 | 0.1699 | Yes |
| 9 | IDH2 | isocitrate dehydrogenase (NADP(+)) 2 [Source:HGNC Symbol;Acc:HGNC:5383] | 967 | 0.525 | 0.1732 | Yes |
| 10 | ACOT8 | acyl-CoA thioesterase 8 [Source:HGNC Symbol;Acc:HGNC:15919] | 1088 | 0.500 | 0.1824 | Yes |
| 11 | GLUD1 | glutamate dehydrogenase 1 [Source:HGNC Symbol;Acc:HGNC:4335] | 1279 | 0.462 | 0.1847 | Yes |
| 12 | GOT2 | glutamic-oxaloacetic transaminase 2 [Source:HGNC Symbol;Acc:HGNC:4433] | 1308 | 0.457 | 0.1994 | Yes |
| 13 | ALDH18A1 | aldehyde dehydrogenase 18 family member A1 [Source:HGNC Symbol;Acc:HGNC:9722] | 1402 | 0.443 | 0.2086 | Yes |
| 14 | SHMT2 | serine hydroxymethyltransferase 2 [Source:HGNC Symbol;Acc:HGNC:10852] | 1518 | 0.426 | 0.2154 | Yes |
| 15 | GLUD2 | glutamate dehydrogenase 2 [Source:HGNC Symbol;Acc:HGNC:4336] | 1554 | 0.420 | 0.2283 | Yes |
| 16 | ALDH5A1 | aldehyde dehydrogenase 5 family member A1 [Source:HGNC Symbol;Acc:HGNC:408] | 1661 | 0.405 | 0.2350 | Yes |
| 17 | ME1 | malic enzyme 1 [Source:HGNC Symbol;Acc:HGNC:6983] | 1746 | 0.392 | 0.2430 | Yes |
| 18 | DGLUCY | D-glutamate cyclase [Source:HGNC Symbol;Acc:HGNC:20498] | 1768 | 0.388 | 0.2558 | Yes |
| 19 | SDHB | succinate dehydrogenase complex iron sulfur subunit B [Source:HGNC Symbol;Acc:HGNC:10681] | 1818 | 0.382 | 0.2661 | Yes |
| 20 | DDO | D-aspartate oxidase [Source:HGNC Symbol;Acc:HGNC:2727] | 1839 | 0.380 | 0.2786 | Yes |
| 21 | ME2 | malic enzyme 2 [Source:HGNC Symbol;Acc:HGNC:6984] | 1870 | 0.377 | 0.2902 | Yes |
| 22 | TAT | tyrosine aminotransferase [Source:HGNC Symbol;Acc:HGNC:11573] | 1979 | 0.363 | 0.2953 | Yes |
| 23 | OAT | ornithine aminotransferase [Source:HGNC Symbol;Acc:HGNC:8091] | 2074 | 0.353 | 0.3010 | Yes |
| 24 | QPRT | quinolinate phosphoribosyltransferase [Source:HGNC Symbol;Acc:HGNC:9755] | 2077 | 0.353 | 0.3139 | Yes |
| 25 | MTHFS | methenyltetrahydrofolate synthetase [Source:HGNC Symbol;Acc:HGNC:7437] | 2155 | 0.343 | 0.3207 | Yes |
| 26 | SDHA | succinate dehydrogenase complex flavoprotein subunit A [Source:HGNC Symbol;Acc:HGNC:10680] | 2231 | 0.335 | 0.3273 | Yes |
| 27 | PHYH | phytanoyl-CoA 2-hydroxylase [Source:HGNC Symbol;Acc:HGNC:8940] | 2397 | 0.315 | 0.3261 | Yes |
| 28 | SDHAF3 | succinate dehydrogenase complex assembly factor 3 [Source:HGNC Symbol;Acc:HGNC:21752] | 2409 | 0.313 | 0.3368 | Yes |
| 29 | ASS1 | argininosuccinate synthase 1 [Source:HGNC Symbol;Acc:HGNC:758] | 2466 | 0.307 | 0.3438 | Yes |
| 30 | KYNU | kynureninase [Source:HGNC Symbol;Acc:HGNC:6469] | 2502 | 0.303 | 0.3523 | Yes |
| 31 | GAD1 | glutamate decarboxylase 1 [Source:HGNC Symbol;Acc:HGNC:4092] | 2534 | 0.299 | 0.3610 | Yes |
| 32 | FTCD | formimidoyltransferase cyclodeaminase [Source:HGNC Symbol;Acc:HGNC:3974] | 3064 | 0.244 | 0.3288 | No |
| 33 | HAAO | "3-hydroxyanthranilate 3,4-dioxygenase [Source:HGNC Symbol;Acc:HGNC:4796]" | 3209 | 0.229 | 0.3260 | No |
| 34 | SUCLA2 | succinate-CoA ligase ADP-forming subunit beta [Source:HGNC Symbol;Acc:HGNC:11448] | 3228 | 0.228 | 0.3331 | No |
| 35 | FPGS | folylpolyglutamate synthase [Source:HGNC Symbol;Acc:HGNC:3824] | 3489 | 0.203 | 0.3203 | No |
| 36 | ASPA | aspartoacylase [Source:HGNC Symbol;Acc:HGNC:756] | 3610 | 0.192 | 0.3181 | No |
| 37 | KYAT3 | kynurenine aminotransferase 3 [Source:HGNC Symbol;Acc:HGNC:33238] | 4128 | 0.143 | 0.2831 | No |
| 38 | ME3 | malic enzyme 3 [Source:HGNC Symbol;Acc:HGNC:6985] | 4613 | 0.104 | 0.2492 | No |
| 39 | AASDHPPT | aminoadipate-semialdehyde dehydrogenase-phosphopantetheinyl transferase [Source:HGNC Symbol;Acc:HGNC:14235] | 5047 | 0.069 | 0.2181 | No |
| 40 | GGT1 | gamma-glutamyltransferase 1 [Source:HGNC Symbol;Acc:HGNC:4250] | 5177 | 0.058 | 0.2101 | No |
| 41 | SLC25A12 | solute carrier family 25 member 12 [Source:HGNC Symbol;Acc:HGNC:10982] | 5506 | 0.032 | 0.1857 | No |
| 42 | FH | fumarate hydratase [Source:HGNC Symbol;Acc:HGNC:3700] | 5729 | 0.013 | 0.1689 | No |
| 43 | DHFR | dihydrofolate reductase [Source:HGNC Symbol;Acc:HGNC:2861] | 5805 | 0.008 | 0.1634 | No |
| 44 | NIT2 | nitrilase family member 2 [Source:HGNC Symbol;Acc:HGNC:29878] | 6200 | -0.021 | 0.1334 | No |
| 45 | SLC7A11 | solute carrier family 7 member 11 [Source:HGNC Symbol;Acc:HGNC:11059] | 6470 | -0.040 | 0.1139 | No |
| 46 | PRODH | proline dehydrogenase 1 [Source:HGNC Symbol;Acc:HGNC:9453] | 6927 | -0.074 | 0.0811 | No |
| 47 | IDH1 | isocitrate dehydrogenase (NADP(+)) 1 [Source:HGNC Symbol;Acc:HGNC:5382] | 7109 | -0.089 | 0.0703 | No |
| 48 | MTHFD2L | methylenetetrahydrofolate dehydrogenase (NADP+ dependent) 2 like [Source:HGNC Symbol;Acc:HGNC:31865] | 7789 | -0.141 | 0.0226 | No |
| 49 | MTRR | 5-methyltetrahydrofolate-homocysteine methyltransferase reductase [Source:HGNC Symbol;Acc:HGNC:7473] | 8087 | -0.162 | 0.0054 | No |
| 50 | ACLY | ATP citrate lyase [Source:HGNC Symbol;Acc:HGNC:115] | 8173 | -0.169 | 0.0051 | No |
| 51 | GLS | glutaminase [Source:HGNC Symbol;Acc:HGNC:4331] | 8423 | -0.187 | -0.0074 | No |
| 52 | NR1H4 | nuclear receptor subfamily 1 group H member 4 [Source:HGNC Symbol;Acc:HGNC:7967] | 8481 | -0.192 | -0.0047 | No |
| 53 | OGDH | oxoglutarate dehydrogenase [Source:HGNC Symbol;Acc:HGNC:8124] | 8503 | -0.194 | 0.0008 | No |
| 54 | GCLC | glutamate-cysteine ligase catalytic subunit [Source:HGNC Symbol;Acc:HGNC:4311] | 8534 | -0.196 | 0.0058 | No |
| 55 | HAL | histidine ammonia-lyase [Source:HGNC Symbol;Acc:HGNC:4806] | 9053 | -0.236 | -0.0259 | No |
| 56 | LIPF | "lipase F, gastric type [Source:HGNC Symbol;Acc:HGNC:6622]" | 9074 | -0.238 | -0.0186 | No |
| 57 | PCK1 | phosphoenolpyruvate carboxykinase 1 [Source:HGNC Symbol;Acc:HGNC:8724] | 9100 | -0.239 | -0.0117 | No |
| 58 | SLC25A32 | solute carrier family 25 member 32 [Source:HGNC Symbol;Acc:HGNC:29683] | 9170 | -0.246 | -0.0080 | No |
| 59 | GAD2 | glutamate decarboxylase 2 [Source:HGNC Symbol;Acc:HGNC:4093] | 9249 | -0.252 | -0.0047 | No |
| 60 | ALDH1L1 | aldehyde dehydrogenase 1 family member L1 [Source:HGNC Symbol;Acc:HGNC:3978] | 9302 | -0.256 | 0.0007 | No |
| 61 | SHMT1 | serine hydroxymethyltransferase 1 [Source:HGNC Symbol;Acc:HGNC:10850] | 9727 | -0.294 | -0.0214 | No |
| 62 | SLC19A1 | solute carrier family 19 member 1 [Source:HGNC Symbol;Acc:HGNC:10937] | 9931 | -0.315 | -0.0256 | No |
| 63 | MTHFD1 | "methylenetetrahydrofolate dehydrogenase, cyclohydrolase and formyltetrahydrofolate synthetase 1 [Source:HGNC Symbol;Acc:HGNC:7432]" | 10249 | -0.346 | -0.0375 | No |
| 64 | GCLM | glutamate-cysteine ligase modifier subunit [Source:HGNC Symbol;Acc:HGNC:4312] | 10439 | -0.366 | -0.0387 | No |
| 65 | DLST | dihydrolipoamide S-succinyltransferase [Source:HGNC Symbol;Acc:HGNC:2911] | 10634 | -0.386 | -0.0395 | No |
| 66 | OGDHL | oxoglutarate dehydrogenase L [Source:HGNC Symbol;Acc:HGNC:25590] | 10754 | -0.399 | -0.0340 | No |
| 67 | IDH3B | isocitrate dehydrogenase (NAD(+)) 3 non-catalytic subunit beta [Source:HGNC Symbol;Acc:HGNC:5385] | 11304 | -0.472 | -0.0593 | No |
| 68 | GLS2 | glutaminase 2 [Source:HGNC Symbol;Acc:HGNC:29570] | 11547 | -0.507 | -0.0594 | No |
| 69 | ALDH4A1 | aldehyde dehydrogenase 4 family member A1 [Source:HGNC Symbol;Acc:HGNC:406] | 12080 | -0.614 | -0.0781 | No |
| 70 | L2HGDH | L-2-hydroxyglutarate dehydrogenase [Source:HGNC Symbol;Acc:HGNC:20499] | 12208 | -0.648 | -0.0640 | No |
| 71 | MTHFD2 | "methylenetetrahydrofolate dehydrogenase (NADP+ dependent) 2, methenyltetrahydrofolate cyclohydrolase [Source:HGNC Symbol;Acc:HGNC:7434]" | 12239 | -0.656 | -0.0420 | No |
| 72 | ADSS2 | adenylosuccinate synthase 2 [Source:HGNC Symbol;Acc:HGNC:292] | 12376 | -0.701 | -0.0266 | No |
| 73 | GLUL | glutamate-ammonia ligase [Source:HGNC Symbol;Acc:HGNC:4341] | 12527 | -0.762 | -0.0101 | No |
| 74 | PRODH2 | proline dehydrogenase 2 [Source:HGNC Symbol;Acc:HGNC:17325] | 12847 | -1.055 | 0.0041 | No |
Table: GSEA details [plain text format]

  

Fig 2: GOBP\_DICARBOXYLIC\_ACID\_METABOLIC\_PROCESS      
 Blue-Pink O' Gram in the Space of the Analyzed GeneSet

  

Fig 3: GOBP\_DICARBOXYLIC\_ACID\_METABOLIC\_PROCESS: Random ES distribution      
 Gene set null distribution of ES for **GOBP\_DICARBOXYLIC\_ACID\_METABOLIC\_PROCESS**
